# Extending the paleontology–biogeography reciprocity with SDMs: Exploring models and data in reducing fossil taxonomic uncertainty

**DOI:** 10.1371/journal.pone.0194725

**Published:** 2018-03-28

**Authors:** Anderson Aires Eduardo, Pablo Ariel Martinez, Sidney Feitosa Gouveia, Franciely da Silva Santos, Wilcilene Santos de Aragão, Jennifer Morales-Barbero, Leonardo Kerber, Alexandre Liparini

**Affiliations:** 1 PIBiLab – Laboratório de Pesquisa Integrativa em Biodiversidade / Integrative Research on Biodiversity Lab, Federal University of Sergipe, Aracajú, State of Sergipe, Brazil; 2 Department of Biology, Federal University of Sergipe, Aracajú, State of Sergipe, Brazil; 3 Department of Ecology, Federal University of Sergipe, Aracajú, State of Sergipe, Brazil; 4 Unit of Ecology, Faculty of Biology, University of Salamanca, Salamanca, C.U. Miguel de Unamuno, Spain; 5 CAPPA – Centro de Apoio à Paleontologia da Quarta Colônia, Federal University of Santa Maria, São João do Polêsine, State of Rio Grande do Sul, Brazil; Swedish Museum of Natural History, SWEDEN

## Abstract

Historically, studies aimed at prospecting and analyzing paleontological and neontological data to investigate species distribution have developed separately. Research at the interface between paleontology and biogeography has shown a unidirectional bias, mostly focusing on how paleontological information can aid biogeography to understand species distribution through time. However, the modern suit of techniques of ecological biogeography, particularly species distribution models (SDM), can be instrumental for paleontologists as well, improving the biogeography-paleontology interchange. In this study, we explore how to use paleoclimatic data and SDMs to support paleontological investigation regarding reduction of taxonomic uncertainty. Employing current data from two neotropical species (*Lagostomus maximus* and *Myocastor coipus*), we implemented SDMs and performed model validation comparing hindcasts with dated fossil occurrences (~14k and ~20k years back present, respectively). Finally, we employed the hindcasting process for two South American fossil records of a misidentified species of caiman (*Caiman* sp.) to show that *C*. *latirostris* is the most likely species identity of these fossils (among four candidate species: *C*. *latirostris*, *C*. *yacare*, *C*. *crocodilus*, and *Melanosuchus niger*). Possible limitations of the approach are discussed. With this strategy, we have shown that current developments in biogeography research can favour paleontology, extending the (biased) current interchange between these two scientific disciplines.

## Introduction

Integration of scientific fields profoundly benefits the bodies of knowledge involved and promotes the discovery of novel solutions to old and new questions. After a long history of separate development, the study of distribution, evolution and diversity of species have been invigorated by a recent integration of paleontological and neontological approaches [1,2]. In the beginning, this interchange mostly focused on macroevolutionary research through development of methods to resolve evolutionary relationships of living and extinct lineages using biogeographic data [3]. More recently, however, integration between paleontology and ecological biogeography–paleobiogeography–has flourished as an approach to investigate patterns of- and processes affecting- species distribution through time [4–7]. A central focus of this emerging field has been to understand the effect of climatic shifts and species interactions on past species distribution and extinction [4–13].

An important tool in this modern biogeography–paleontology interchange has been the suite of techniques comprising species distribution modelling (SDM). This integration has boosted biogeography and paleobiogeography with a more in-depth understanding of temporal changes in species distributions and their interactions with environmental changes through time, while also relying on more powerful methods of statistical and ecological modelling [4,5,13]. Furthermore, the increasing development and availability of paleoclimatic data have improved temporal range and resolution of SDM applications, including thousand-year basis sequences (e.g., [14]). This availability of data at high spatial and temporal resolution, together with SDM techniques, opens more opportunities for this interchange between paleontology and biogeography than previously appreciated.

So far, however, the recent developments in the paleontology–biogeography interchange have been largely unidirectional, in the sense that they have focused largely on how paleontological information–through fossil records–can aid biogeographers to understand species distributions through time [4,5,13,15]. However, this suite of techniques of ecological biogeography, particularly SDM, can be instrumental for paleontologists as well, but have remained poorly explored (e.g., [16]). For example, one important problem for paleontologists is uncertainty in the taxonomic identification due to the fragmentary nature of fossil records [17]. SDM can help to at least lessen this problem. That is, provided that the candidate taxa of the fossil exhibit distinguishable environmental preferences, SDM can discriminate between niches, and then assign an ambiguous fossil to one (or a few) most likely species according to their actual potential niches. Note though that we do not mean that SDM can be used to taxonomically identify a fossil–which should be a task for paleontological taxonomists. Instead, SDM can be useful to reduce taxonomic uncertainty when niches of involved species are distinguishable. Although simple, we argue this strategy can be a useful element in the paleontologist toolkit.

To assist fossil identification, however, SDM will depend on paleoclimatic data layers that are reliable and that match fossils’ ages, as well as on accurate model estimation. These constitutes critical aspects for the approach we advocate here. To address these requirements, dated fossils of extant species can help to assess the reliability of paleoclimatic data layers and the model accuracy. These fossils can be used to validate hindcast models that are built from records of the present. If models can accurately predict the species occurrence in the fossil location at the period corresponding to the fossils age, then this model can correctly discriminate the climatic settings that characterize the species niche. Consequently, we can use the reverse reasoning to use competing models of candidate species to discriminate among each other and assign the fossil to the more likely species. Here, it is inevitable to rely on the inherent assumptions of SDMs [18]. Thus, this step explicitly assumes (i) that the species distribution is in equilibrium with the environment, i.e., that the species currently occupies all those areas suitable for it [16], and (ii) that climatic niches are conserved through recent geological time [18]. (additional caveats, inherent to the proposed approach, are presented in the Discussion).

Thereby, in this study our first aim is to assess the reliability of the paleoclimatic data in the context of high temporal and spatial resolutions [19], as well as model accuracy to predict the spatial and temporal positions of known fossils. To do this, we use two living rodent species that have well-known current distributions and that are represented by dated fossils that were found outside of their current distribution. We then built upon this by using an SDM approach and assessed its use in reducing taxonomic uncertainty of fossils identified up to taxonomic level of genus. Using two fossil occurrences of a caiman (*Caiman* sp.), we employ SDM to address the question of which species (among four candidate species) is the more likely to be represented by the fossils, according to their climatic preferences.

## Material and methods

### Data compilation

We implemented SDMs for two rodent species, *Lagostomus maximus*, *Myocastor coypus*, and four caiman species, *Caiman yacare*, *Caiman latirostris*, *Caiman corcodilus*, *Melanosuchus niger* (see section “Evaluating paleoclimatic data and models” for ecological and paleontological details). Occurrence records of the current distribution of these six species were obtained from the online platforms Species Link, available at www.splink.org.br, and Global Biodiversity Information Facility (GBIF), available at www.gbif.org. For the caiman species, we supplemented our dataset with records found in the literature (see Table C in S1 File), totalizing 315 records (91 for *C*. *yacare*, 92 for *C*. *latirostris*, 105 for *C*. *c*. *crocodilus* and 27 for *Melanosuchus niger*) after data cleaning to remove duplicates and suspicious records (i.e., records with dubious taxonomic identification and points of occurrence out of the IUCN species range). Thus, we certify that uncertainties related to the current distribution of species will not affect the models.

To describe the climatic settings of the species distributions, we used four non-collinear (i.e., r < 0.7) bioclimatic variables (derived from monthly precipitation and temperature) for the study area (South America) that, in addition, are inherently informative of the major climatic changes undertaken in Neotropics during the later glacial cycle [20–22]. Variables included mean temperature of the warmest and the coldest quarters (Bio10 and Bio11), and total precipitation of the driest and wettest quarters (Bio16 and Bio17). These variables were obtained from [19], which derived from the Hadley Centre Coupled Model (HadCM3) [23], and consist of a sequence of paleoclimatic data layers at a spatial resolution of 2.5’ (~25 km^2^) and a temporal resolution of 1,000 years (1 kyr), from the present back to 130,000 years before present (or 130 kyr BP). The non-occurrence of non-analogue climates was verified through Maxent outputs for Clamping, MESS and MoD (see S4–S7 Figs).

### Species distribution models

We modeled the species distribution with three algorithms, Maxent [24], Random Forest (RF; [25]) and Generalized Linear Models (GLM; [26]). The two former are machine-learning algorithms, whereas the latter is a regression-based process [27]. We partitioned the data set in order to evaluate the models, using 75% as the training set and 25% as a test set. After that, we employed the full data set for model fitting and projections. Following other authors (e.g. [28–30]) we employed randomly distributed points as pseudo-absences (without overlap with the species occurrences) for RF and GLM algorithms. For Maxent we drew background points at random from South America. We then extracted the climatic conditions of each of these localities to perform the modelling procedures. To construct the models with Maxent, we used 1000 iterations, 1000 background points, a regularization value of 1, and a convergence threshold of 1x10^-5^ (see recommendations by [31]). For the RF model, the number of trees used determines the model accuracy, so we chose 500 after ensuring this was sufficient for the model to stabilize. Finally, we use GLMs, with a binomial distribution and logistic link. Only linear and quadratic features were allowed in the models.

Model performance was evaluated through cross-validation, using the Area Under the Receiver Operating Characteristic curve, or AUC [32], and the True Skill Statistic (TSS) [33]. The AUC measures model accuracy using the ratio between the rate of correctly predicted presences (sensitivity) and the rate of incorrectly predicted absences (1 minus specificity). TSS compares the number of correct projections (minus those attributable to random guessing) to a hypothetical set of correct projections. These procedures were repeated 50 times, with resampling of training and test sets for each iteration. We used the Dismo [34] and randomForest [35] packages in R (version 3.3.1).

Our aim was to evaluate the use of SDM as a tool to assess past species distribution at specific ages in the geological past, and to reconstruct past species distributions and reduce uncertainties in taxonomic identification. Therefore, for the paleoclimatic data and model evaluation (performed with the two rodent species), we compared differences in model performances through the differences of the models’ suitability (or projected environmental suitability, ranging from 0 to 1) observed at the location of the fossil occurrence. Conceptually, suitability can be interpreted as a surrogate measure of the probability of occurrence of a species in an area [24,36]. Then, to address the problem of fossils’ taxonomic uncertainty (among the four caiman species), we used the approach to assess which of the four candidate species is more likely to be represented by the fossil specimens. For each species, we used the minimum suitability value observed at the occurrence points (extracted from the projections for 0 kyr, i.e., current age) to help us infer the reliability of species occurrence at the fossil site. In addition, we calculated the pairwise correlation–through Pearson’s correlation–among the distribution maps obtained from each algorithm for each species at each period to assess their concordance. Models were generally correlated (Peason’s r > 0.7, with the exception of *C*. *yacare*, which had greater reduction in the suitability in hindcast models; see Table A-Table M in S1 File, S1–S3 Figs). Therefore, in the main text, we present the results from Maxent, the results from the other algorithms are in the Supporting Information material.

### Evaluating paleoclimatic data and models

The success of our approach rely directly on accurate models and data. So, we firstly assessed the reliability of paleoclimatic data and models in correctly predicting the species occurrence in the fossil location and the period corresponding to the fossils’ age. To this end, we selected species that had well-known geographic limits, a large number of georeferenced records that covered their entire distribution, and absolute dated fossil occurrences with known geographical location (coordinates).

The first species case was *Lagostomus maximus* (Rodentia, Chinchillidae), a native rodent from South America. Its current distribution includes central and northern Argentina, southern Paraguay and southern and eastern Bolivia [37,38]. Fossil records of *L*. *maximus* have been identified from the late Pleistocene onwards [39–41]. This species is thought to have been locally extinct from Uruguay and the extreme southern portion of Brazil since the early Holocene, with last occurrence records in the late Pleistocene [40,41]. We used the fossil material of *L*. *maximus* that was found in the Dolores formation, Uruguay (Table A in S1 File), and was dated to the late Pleistocene, between 13,898 and 13,941 years BP (calibrated dating; [40]). This location is marginally outside the current distribution of the species. We built SDMs from the current distribution and projected them between 13 and 14 kyr BP, with the expectation that models for this time period should predict the occurrence of *L*. *maximus* at this location.

The second species was *Myocastor coypus* (Rodentia, Echimyidae), which is a rodent native to southern South America, distributed throughout Argentina, Uruguay, Paraguay, Chile, Bolivia and southern Brazil [42]. Fossil occurrences of this species have been found from the late Pleistocene into the Holocene [43,44], comprising areas in northern Argentina, Uruguay, southern Bolivia (inside its current distribution), and southern, southeastern and northeastern Brazil (areas outside its current distribution) [44–46]. We used a fossil occurrence from northeastern Brazil (~2000 km from the current distribution of the species), which is dated to 19,980–20,250 years BP (calibrated dating; [47]) (Table A in S1 File). Here, we also evaluated the models’ effectiveness in predicting the species occurrence at the true location and age (between 19 and 20 kyr BP). As this locality is far outside the species’ current distribution, if models also predict the spatial and temporal position of this fossil accurately, it would indicate that the approach is effective in describing geographical distributions of species at high spatial and temporal resolutions, and thus in discriminating among species with inconclusive identification but with different climatic niches.

### Addressing fossils’ taxonomic uncertainty

To apply the above reasoning in a real case of species misidentification, we choose the case of the fossils of *Caiman* sp. (Reptilia, Alligatoridae). This genus of alligators has three living species [48]. *C*. *yacare* occur at the central-southern South America, including Bolivia, Paraguay, north Argentina and central-western Brazil (Crocodile Specialist Group, 1996a). *C*. *latirostris* predominates along the Atlantic coast of South America, from northeastern Brazil to Uruguay, and in northeastern Argentina, Paraguay, eastern Bolivia and central-western Brazil [49]. *C*. *crocodilus* is distributed from Guatemala to southern Amazonia and central-western Brazil [50]. This species comprises three subspecies, *C*. *c*. *chiapasius* (from Central America), *C*. *c*. *fuscus* (from Central America and northwestern South America) and *C*. *c*. *crocodilus*, which diverged from its conspecifics at 5.5 myr at least, and is broadly distributed in South America [51]. Additionally, we included in the analysis the species *Melanosuchus niger*, because it is morphologically and phylogenetically closely related to the *Caiman* genus. This species is found in the Amazon River basin; occurring in northern Bolivia, east of Peru and Ecuador, and in southern Colombia and Guyana [52].

The oldest fossil record of the genus *Caiman* is from the middle Miocene, of Colombia [53]. The late Miocene fossil record of this genus is sparse and the material is poorly preserved [54], which precludes conclusive identification. Although distributed throughout most of South America (the southern portion of the continent and central and northern Amazonia), crocodilian fossils in South America from the Pleistocene that are unambiguously identified to the species level are rare (and identification is often not feasible from the literature) [54]. For this case study, we employed SDM to aid in reducing taxonomic uncertainty of two fossil specimens. One fossil specimen, identified to the genus level, was found in Ioiô cave (Table B in S1 File), Iaraquara municipality, Bahia, northeastern Brazil, and was estimated to be late Pleistocene in age (21,520–22,040 years BP, calibrated dating). A second fossil was excavated in Poço Redondo municipality, Sergipe, northeastern Brazil (Table B in S1 File), and was estimated to be early Holocene (11,068–11,211 years BP, calibrated dating). The specimen was tentatively identified as *C*. *latirostris* based on the current distribution of the species [55]. Here we implemented hindcast models of three Caiman species (*C*. *yacare*, *C*. *latirostris* and *C*. *crocodilus crocodilus*) and *Melanosuchus niger*, based on records of their current distribution and projected to 21 and 11 kyr BP to attempt to assign the fossils’ identity to one of the candidate species.

## Results

### Paleoclimatic data and models

**Fig 1:**
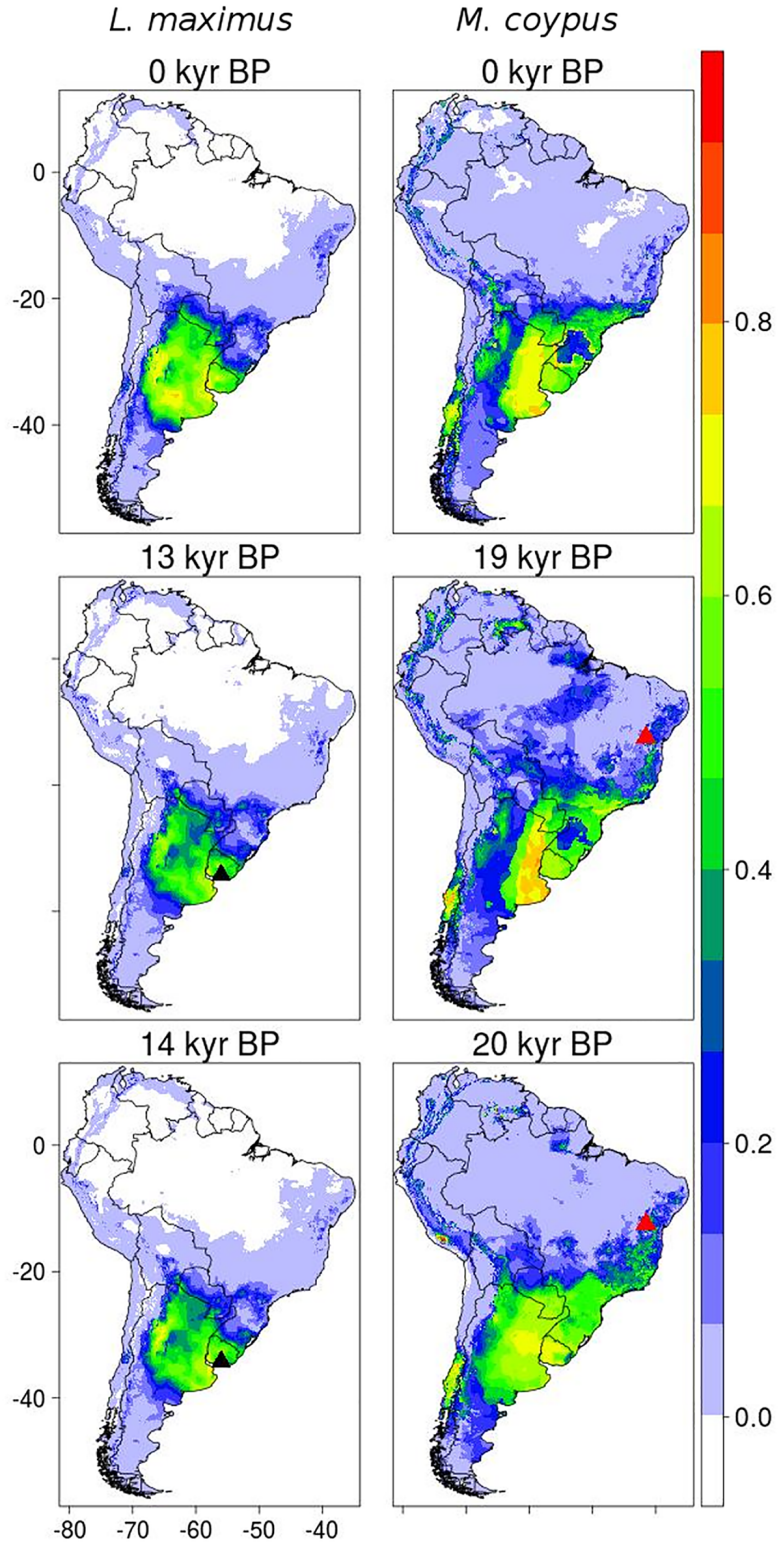
Results of species distribution modeling (SDM) employing Maxent algorithm, for the species *Lagostomus maximus* (left column) and *Myocastor coypus* (right). The suitability projections for current time are showed in continuous scale (between 0 and 1). Triangles represent the coordinates of fossil records.

Model accuracy from Maxent for the rodents *L*. *maximus* and *M*. *coypus* was high (AUC = 0.91 and TSS = 0.84; AUC = 0.93 and TSS = 0.72, respectively), indicating good model fit. The projected distribution of *L*. *maximus* agreed with its distribution described in the literature (Fig 1). Model suitability for the specific location of this species was 0.46 and 0.47 for 13 kyr BP and 14 kyr BP respectively, being 0.17 the lowest suitability observed among occurrence data (from model’s projection for 0 kyr). Thus, the model was able in predicting the fossil temporal and spatial position.

For *M*. *coypus*, where the fossil was recorded ~2,000 km away from the current distribution of the species, models predicted suitable habitat in the region of the fossil record in the past. Model suitability at the geographical location of the fossil was 0.41 and 0.50, for 19 kyr BP and 20 kyr BP, respectively, thus being capable in capturing the spatial and temporal position of the fossil (currently, lowest suitability among occurrence points was 0.04). Together with the previous result, this shows that the data and the modeling approach employed are valid for our purposes.

### Fossils’ taxonomic uncertainty

**Fig 2:**
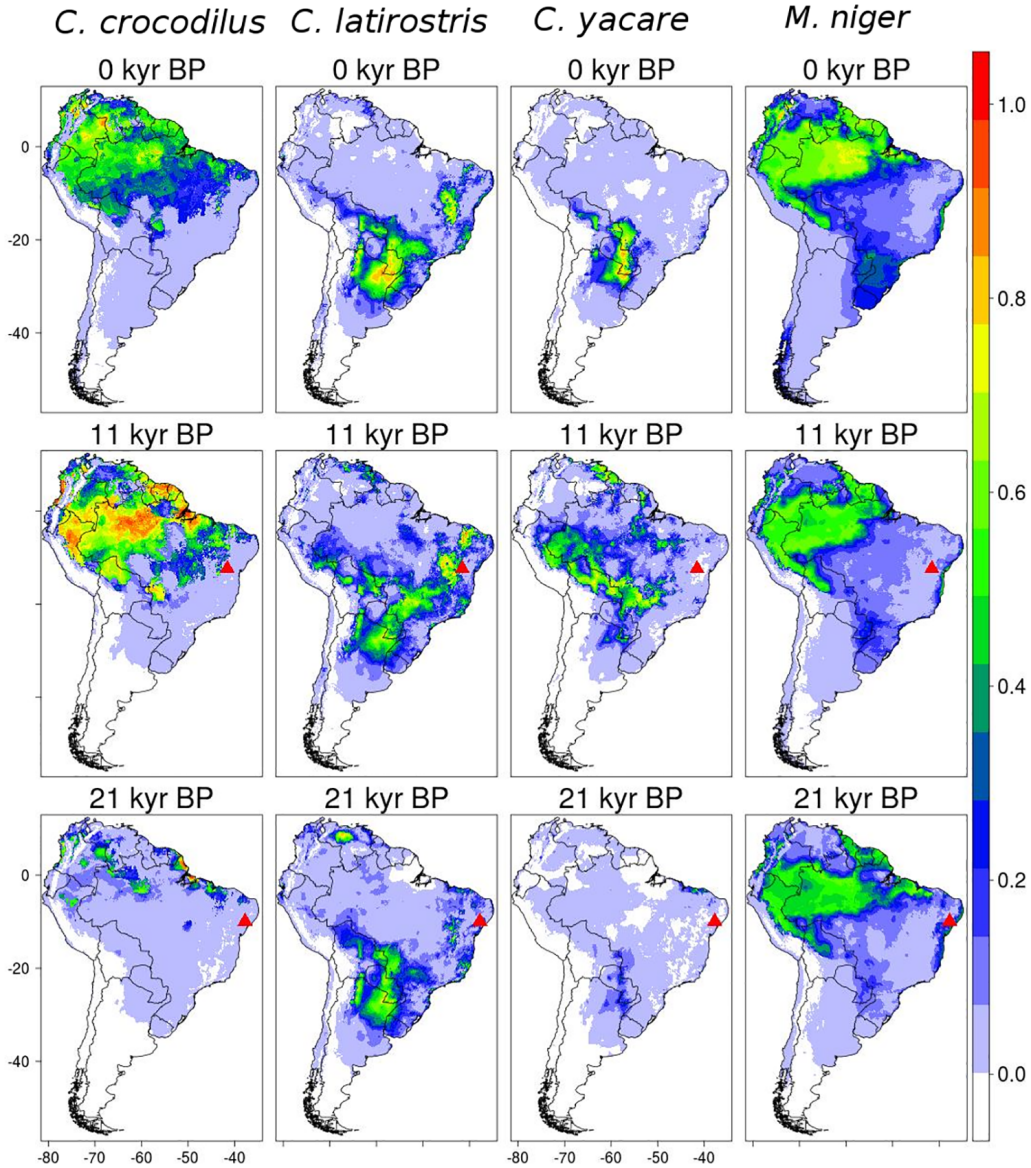
Results of Maxent algorithm for the caiman species (*C*. *c*. *crocodilus*, *C*. *yacare*, *C*. *latirostsris*, and *M*. *niger*). The suitability projections for current time are showed in continuous scale (between 0 and 1). Triangles represent the coordinates of fossil records.

In the caiman case, in which we modeled the distribution of four candidate species for two fossil specimens, we found model accuracies of AUC = 0.84 and TSS = 0.56 for *C*. *c*. *crocodilus*, AUC = 0.85 and TSS = 0.66 for *C*. *latirostris*, AUC = 0.96 and TSS = 0.86 for *C*. *yacare*, and AUC = 0.79 and TSS = 0.64 for *M*. *niger*. The predicted distributions for the present agreed with the species known distribution, and tended to be narrower with the older paleoclimatic data layers (Fig 2). For the fossil from the Ioiô cave, dated to 21,520–22,040 years BP, the hindcast models for 21 kyr BP assigned suitability of 0.01 for *C*. *c*. *crocodilus*, <0.001 for *C*. *yacare*, 0.09 for *C*. *latirostris*, and <0.001 for *M*. *niger*, thus *C*. *latirostris* is the most likely species to be represented by these fossils, according to the models (currently, lowest suitability in occurrence data was 0.14, 0.01, 0.04, 0.35, for the respective species). For the second fossil from Poço Redondo, Sergipe, dated to 11,068–11,211 years BP and tentatively identified as *C*. *latirostris*, the models assigned suitability of <0.001 for *C*. *c*. *crocodilus*, *C*. *yacare* and *M*. *niger*, and 0.04 for *C*. *latirostris*, in agreement with the previous identification (orderly, 0.14, 0.01,0.35, 0.04 were the lowest current suitability in the occurrence data).

## Discussion

We have shown that the models were able to discriminate occurrence locations of the species investigated at the time period corresponding to the fossils ages, irrespective of the particular period, algorithm used, and the potential magnitude of effect the last Pleistocene-Holocene climate changes have had on these species [11, 14, 15]. In the species cases used for paleoclimatic data and model evaluation (i.e., the rodents), the fossils occurrences are associated with different moments throughout the period of major climatic changes in the last 25 kyr [56–58]. That is, whether the past species distributions were coincident or not with their current distribution, and whether they were representative of late interglacial (~6 kyr BP) or the late maximum glacial (~20 kyr BP), in both cases the fossils’ geographic positions were correctly predicted by the models. Therefore, this preliminary assessment of models and paleoclimatic data validates our second and main goal of using the reversal reasoning to reduce fossils’ taxonomic uncertainty.

In the case of the caimans, all three algorithms of the presumed species (*C*. *latirostris*) also projected its occurrence at the location and period of the fossils’ ages, which correspond to the maximum glacial and the beginning of the interglacial period. Although models indicated *C*. *latirostris* as the most likely species to be represented by the fossils, these were located in areas predicted to have relatively low suitability. This may result either from the inherent uncertainties in data and/or models [59,60], or from taphonomic issues associated to fossil transportation. Caimans are closely associated with rivers, which may transport animals’ carcasses away from their occurrence area, which would also explain the poor preservation of the fossil material [54,61,62]. Despite this, we noted that core areas of projected suitability were not at the geographical vicinity of the fossil locations, except for *C*. *latirostris* SDMs (Fig 2). Still, in general, the consensus and success of SDM in reducing the taxonomic uncertainty in this case reinforces the potential of SDM to investigate different problems of past distributions of species [4,5,13].

Despite the increased use of hindcast models with fossil data (reviewed in [4,5,13]), studies have used fossils mostly as ancillary data to either build models or validate them (e.g., [5,8,9,11,15,63–65]). Of course, as SDM has been developed within ecology and biogeography, it is expected that external data and questions related to SDMs have so far served most as subsidiary to these disciplines than otherwise. For example, modelers have included human demographic data to improve current species distribution patterns (reviewed in [27]). Still, this integrative approach–specifically involving fossils–has succeeded in providing critical insights on the general trends of species distribution along recent geological periods, including assessment of ecological interactions and the drivers of species extinctions [1,2,4–13,66]. Nevertheless, few attempts had been made to use of SDM as a tool to address fossil issues such as taxonomic uncertainty (e.g., [67]).

In view of the increased popularity and easiness of SDM implementation, paleontologists can take advantage of the present reasoning to address several problems of species identification and distribution. As high-resolution paleoclimatic reconstructions and fossil dating techniques improve in quality and availability, and SDM becomes more sophisticated, new opportunities to promote more reciprocity between paleontology and biogeography should take place, benefiting paleontology in its different fields. For instance, hypotheses evaluation regarding paleodistributions would be favoured, because fossils currently non-identified to the species level could become informative through the approach we propose here. Investigation of extinct and living species’ duration could also profit, since the age of the first occurrence could be reassessed using information from fossil data otherwise considered of insufficient taxonomic resolution. Investigation of the morphology of extinct species is another paleontological field that could benefit from the approach outlined here. By increasing the fossil information on a particular species, the more consistent the morphological inferences should be, specially in cases of species with scarce fossil data. In this same sense, studies of morphological variations across geographic space could benefit from new information obtained from occurrences of fossils with improved taxonomic identity.

The major caveats of the use of SDM in paleoecology or paleontology include the implicit assumption of niche stability through time ([21], but see [6]) and the equilibrium of species distribution with climate [68], especially regarding niche transferability to different locations and periods [5,15]. However, this problem pervades the whole field of ecological modeling, including paleobiogeography, specifically due to the difficulty of validating past models (e.g., with fossils). In this regard, this feature adds uncertainty rather than invalidating the models [69,70]. Users should be aware of these limitations, and account for the multiple sources of bias and uncertainty [10]. We can also point out three other limitations of this approach. The first one is the possible existence of unsampled species in the analyses. As the approach deals with fossil material, it is possible that extinct, unknown species co-occurred with the species evaluated. A similar issue is that of the existence of known syntopic species, which increases the possibility of overlapping habitat suitability from models of different species. This certainly increases with the diversity of taxonomic group investigated. Both cases will create a confounding effect between the co-occurring species, this reducing the discriminatory ability of the SDM. Either way, the approach will still be capable of reducing the uncertainty to fewer candidate species, to which other discriminatory strategy can be employed.

A third limitation is the requirement of a minimum dataset of occurrences to estimate the climate preferences of the species. Because the approach focuses on fossils–which are fortuitous occurrence data and are the only source of information available for extinct species–assembling a sufficient number of records of a species from a specific period to build reliable models can be overly complicated (but see [71]). Thus, the approach will be much more effective for fossils of living species, such as those investigated here.

High-resolution paleoclimatic reconstructions have (for now) a limited temporal reach (usually back to the Pleistocene—Holocene transition; [19,21]), and there are serious technical limitations in projecting these climatic reconstructions further back in time (for a discussion regarding climatic variables at the Last Glacial Maximum, see [72]). Therefore, the approach outlined here is better suited for cases of recently extinct or living species, at least for now. In this regard, as this approach is more helpful to paleontologists of recent groups, investigations on these groups should view this reasoning as an opportunity to develop novel questions and insights.

In summary, using SDM, we evaluated the effectiveness of paleoclimatic data at high spatial and temporal resolution in accurately predicting paleodistribution of living species, with known fossil records. In addition, we have shown how this strategy could be useful to reduce taxonomic uncertainty of the fossil specimens, based on the climatic preferences of the candidate species. This strategy represents a further interchange between paleontology and biogeography, with a particular benefit for paleontologist. We highlight the limitations of the approach, related to the possible existence of known or unknown syntopic species, and the dependence of a minimal occurrence dataset to produce fair estimates of species climatic preferences. Notwithstanding, this approach can be well explored by the paleontology of recent groups, which can reward to their biogeography and related fields with fresh insights on species identity and distribution.

## Supporting information

S1 File **Table A: Results for AUC (Area Under the ROC Curve), TSS (True Skill Statistics), and suitability**. Values were extracted from a raster layer, at the coordinates for fossil record of *Lagostomus maximus* (34°16’12.23”S, 55°59’35.82”W and 34°17’30.45”S, 55°55’57.16”W; 13,898–13,941 years BP; Ubila & Rinderknech, 2016) and *Myocastor coypus* (12°23’36.3” S, 41°33’11” W; 19,989–20,250 years BP; Castro et al. 2014). **Table B: Results for AUC (Area Under the ROC Curve), TSS (True Skill Statistics), and suitability**. Values were extracted from a raster layer, at the coordenates for fossil record of *Caiman latirostris* (09°55’37” S, 37°45’13” W; 11,068–11,211 years BP; França et al. 2014) and *Caiman spp*. (12°23’36.3” S, 41°33’11” W; 21,520–22,040 years BP; Castro et al. 2014). **Table C: Occurrence points for Caiman obtained from literature**. These occurrences were added to GBIF data to construct the implemented models. **Table D: Pearson correlation for the suitability maps generated by the three algorithms for *Caiman crocodilus*, 11kyr BP. Table E: Pearson correlation for the suitability maps generated by the three algorithms for *Caiman latirostris*, 11kyr BP. Table F: Pearson correlation for the suitability maps generated by the three algorithms for *Caiman yacare*, 11kyr BP. Table G: Pearson correlation for the suitability maps generated by the three algorithms for *Melanosuchus niger*, 11kyr BP. Table H: Pearson correlation for the suitability maps generated by the three algorithms for *Caiman crocodilus*, 21kyr BP. Table I: Pearson correlation for the suitability maps generated by the three algorithms for *Caiman latirostris*, 21kyr BP. Table J: Pearson correlation for the suitability maps generated by the three algorithms for *Caiman yacare*, 21kyr BP. Table K: Pearson correlation for the suitability maps generated by the three algorithms for *Melanosuchus niger*, 21kyr BP. Table L: Pearson correlation for the suitability maps generated by the three algorithms for *Lagostomus maximus*, 13kyr BP. Table M: Pearson correlation for the suitability maps generated by the three algorithms for *Miocastor coypus*, 21kyr BP**.
(DOC)

S1 Fig Maxent results. Complete results of our simulations with Maxent algorithm, for suitability distribution of *Caiman crocodilus*, *Caiman latirostris*, *Caiman yacare* and *Melanosuchus niger* on the Neotropics. The blue triangle indicates the points where the fossil were recorded.
(TIFF)

S2 Fig Random forest results. Complete results of our simulations with Random Forest algorithm, for suitability distribution of *Caiman crocodilus*, *Caiman latirostris*, *Caiman yacare* and *Melanosuchus niger* on the Neotropics. The blue triangle indicates the points where the fossil were recorded.
(TIFF)

S3 Fig GLM results. Complete results of our simulations with GLM algorithm, for suitability distribution of *Caiman crocodilus*, *Caiman latirostris*, *Caiman yacare* and *Melanosuchus niger* on the Neotropics. The blue triangle indicates the points where the fossil were recorded.
(TIFF)

S4 Fig Climate comparison results. Maxent outputs for Clamping, MESS and MoD, comparing current and 11 kyr BP climates. Results show that do not occur non-analogue climates for such time period, considering the environmental data employed (mean temperature of the warmest and the coldest quarters, and total precipitation of the driest and wettest quarters).
(TIFF)

S5 Fig Climate comparison results. Maxent outputs for Clamping, MESS and MoD, comparing current and 21 kyr BP climates. Results show that do not occur non-analogue climates for such time period, considering the environmental data employed (mean temperature of the warmest and the coldest quarters, and total precipitation of the driest and wettest quarters).
(TIFF)

S6 Fig Climate comparison results. Maxent outputs for Clamping, MESS and MoD, comparing current and 13 kyr BP climates. Results show that do not occur non-analogue climates for such time period, considering the environmental data employed (mean temperature of the warmest and the coldest quarters, and total precipitation of the driest and wettest quarters).
(TIFF)

S7 Fig Climate comparison results. Maxent outputs for Clamping, MESS and MoD, comparing current and 20 kyr BP climates. Results show that do not occur non-analogue climates for such time period, considering the environmental data employed (mean temperature of the warmest and the coldest quarters, and total precipitation of the driest and wettest quarters).
(TIFF)
