# Supplementary material for: Extending the paleontology–biogeography reciprocity with SDMs: Exploring models and data in reducing fossil taxonomic uncertainty
Source: PLoS One. 2018 Mar 28;13(3):e0194725. doi: 10.1371/journal.pone.0194725 (PMC5874039; doi:10.1371/journal.pone.0194725)
Supplement: S1 File — Table A: Results for AUC (Area Under the ROC Curve), TSS (True Skill Statistics), and suitability. Values were extracted from a raster layer, at the coordinates for fossil record of Lagostomus maximus (34°16’12.23”S, 55°59’35.82”W and 34°17’30.45”S, 55°55’57.16”W; 13,898–13,941 years BP; Ubila & Rinderknech, 2016) and Myocastor coypus (12°23’36.3” S, 41°33’11” W; 19,989–20,250 years BP; Castro et al. 2014). Table B: Results for AUC (Area Under the ROC Curve), TSS (True Skill Statistics), and suitability. Values were extracted from a raster layer, at the coordenates for fossil record of Caiman latirostris (09°55’37” S, 37°45’13” W; 11,068–11,211 years BP; França et al. 2014) and Caiman spp. (12°23’36.3” S, 41°33’11” W; 21,520–22,040 years BP; Castro et al. 2014). Table C: Occurrence points for Caiman obtained from literature. These occurrences were added to GBIF data to construct the implemented models. Table D: Pearson correlation for the suitability maps generated by the three algorithms for Caiman crocodilus, 11kyr BP. Table E: Pearson correlation for the suitability maps generated by the three algorithms for Caiman latirostris, 11kyr BP. Table F: Pearson correlation for the suitability maps generated by the three algorithms for Caiman yacare, 11kyr BP. Table G: Pearson correlation for the suitability maps generated by the three algorithms for Melanosuchus niger, 11kyr BP. Table H: Pearson correlation for the suitability maps generated by the three algorithms for Caiman crocodilus, 21kyr BP. Table I: Pearson correlation for the suitability maps generated by the three algorithms for Caiman latirostris, 21kyr BP. Table J: Pearson correlation for the suitability maps generated by the three algorithms for Caiman yacare, 21kyr BP. Table K: Pearson correlation for the suitability maps generated by the three algorithms for Melanosuchus niger, 21kyr BP. Table L: Pearson correlation for the suitability maps generated by the three algorithms for Lagostomus maximus, 13kyr BP [file pone.0194725.s001.doc]

**Supporting information**

**S1 File: Table A: Results for AUC (Area Under the ROC Curve), TSS (True Skill Statistics), and suitability.** Values were extracted from a raster layer, at the coordenates for fossil record of *Lagostomus maximus* (34º16’12.23’’S, 55º59’35.82’’W and 34º17’30.45’’S, 55º55’57.16’’W; 13,898-13,941 years BP; Ubila & Rinderknech, 2016) and *Myocastor coypus* (12º23’36.3’’ S, 41º33’11’’ W; 19,989-20,250 years BP; Castro et al. 2014).

**S1 File: Table B: Results for AUC (Area Under the ROC Curve), TSS (True Skill Statistics), and suitability.** Values were extracted from a raster layer, at the coordenates for fossil record of *Caiman latirostris* (09º55’37’’ S, 37º45’13’’ W; 11,068-11,211 years BP; França et al. 2014) and *Caiman spp.* ( 12º23’36.3’’ S, 41º33’11’’ W; 21,520-22,040 years BP; Castro et al. 2014).

**S1 File: Table C**: Occurrence points for Caiman obtained from literature. This occurrences were added to GBIF data to construct the implemented models.

**S1 File: Table D:**  **Pearson correlation for the suitability maps generated by the three algorithms for *Caiman crocodilus*, 11kyr BP.**

**S1 File: Table E:**  **Pearson correlation for the suitability maps generated by the three algorithms for *Caiman latirostris*, 11kyr BP.**

**S1 File: Table F:**  **Pearson correlation for the suitability maps generated by the three algorithms for *Caiman yacare*, 11kyr BP.**

**S1 File: Table G: Pearson correlation for the suitability maps generated by the three algorithms for *Melanosuchus niger*, 11kyr BP.**

**S1 File: Table H:**  **Pearson correlation for the suitability maps generated by the three algorithms for *Caiman crocodilus*, 21kyr BP.**

**S1 File: Table I: Pearson correlation for the suitability maps generated by the three algorithms for *Caiman latirostris*, 21kyr BP.**

**S1 File: Table J: Pearson correlation for the suitability maps generated by the three algorithms for *Caiman yacare*, 21kyr BP.**

**S1 File: Table K: Pearson correlation for the suitability maps generated by the three algorithms for *Melanosuchus niger*, 21kyr BP.**

**S1 File: Table L: Pearson correlation for the suitability maps generated by the three algorithms for *Lagostomus maximus*, 13kyr BP.**

**S1 File: Table M: Table. Pearson correlation for the suitability maps generated by the three algorithms for *Miocastor coypus*, 20kyr BP.**
