# Supplementary figures and images for: Extending the paleontology–biogeography reciprocity with SDMs: Exploring models and data in reducing fossil taxonomic uncertainty
Source: PLoS One. 2018 Mar 28;13(3):e0194725. doi: 10.1371/journal.pone.0194725 (PMC5874039; doi:10.1371/journal.pone.0194725)

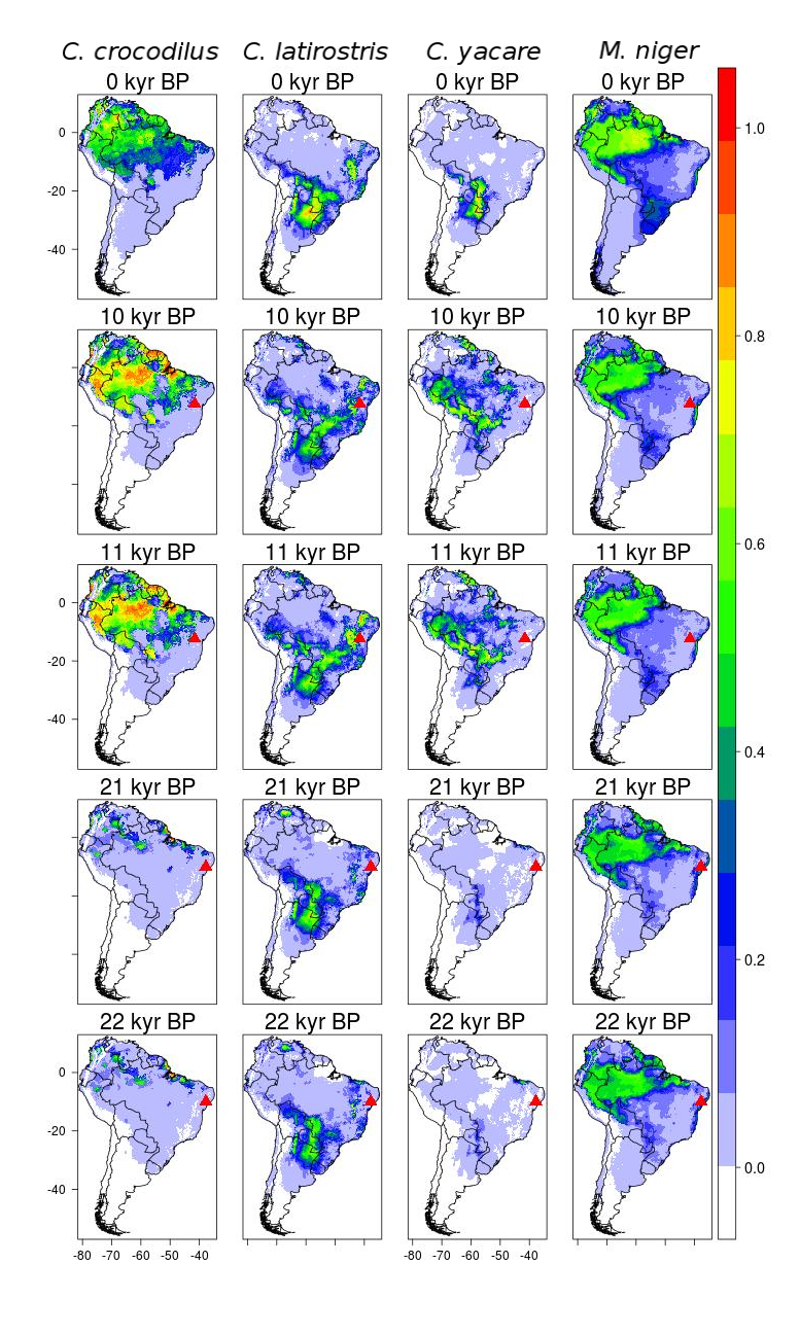

Supplement: S1 Fig — Complete results of our simulations with Maxent algorithm, for suitability distribution of Caiman crocodilus, Caiman latirostris, Caiman yacare and Melanosuchus niger on the Neotropics. The blue triangle indicates the points where the fossil were recorded. (TIFF) [file pone.0194725.s002.tiff]

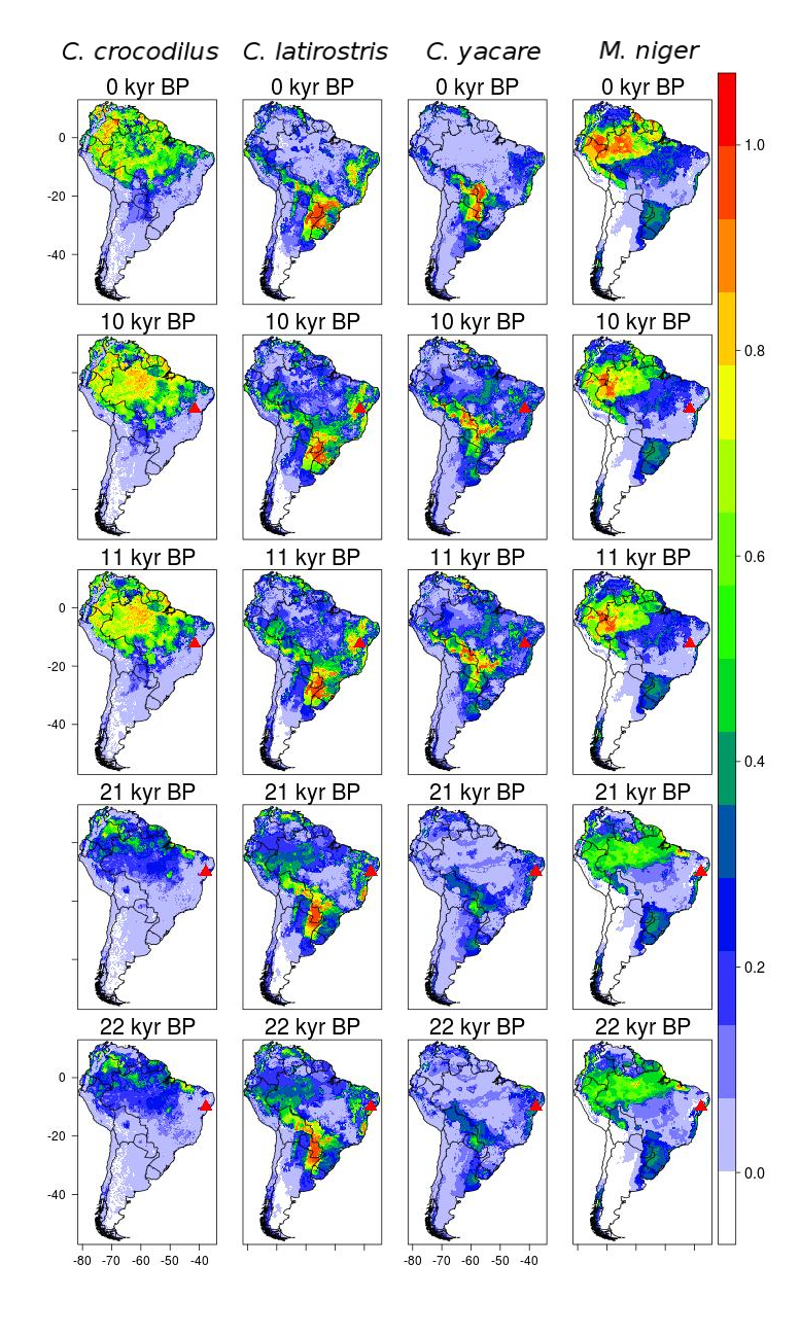

Supplement: S2 Fig — Complete results of our simulations with Random Forest algorithm, for suitability distribution of Caiman crocodilus, Caiman latirostris, Caiman yacare and Melanosuchus niger on the Neotropics. The blue triangle indicates the points where the fossil were recorded. (TIFF) [file pone.0194725.s003.tiff]

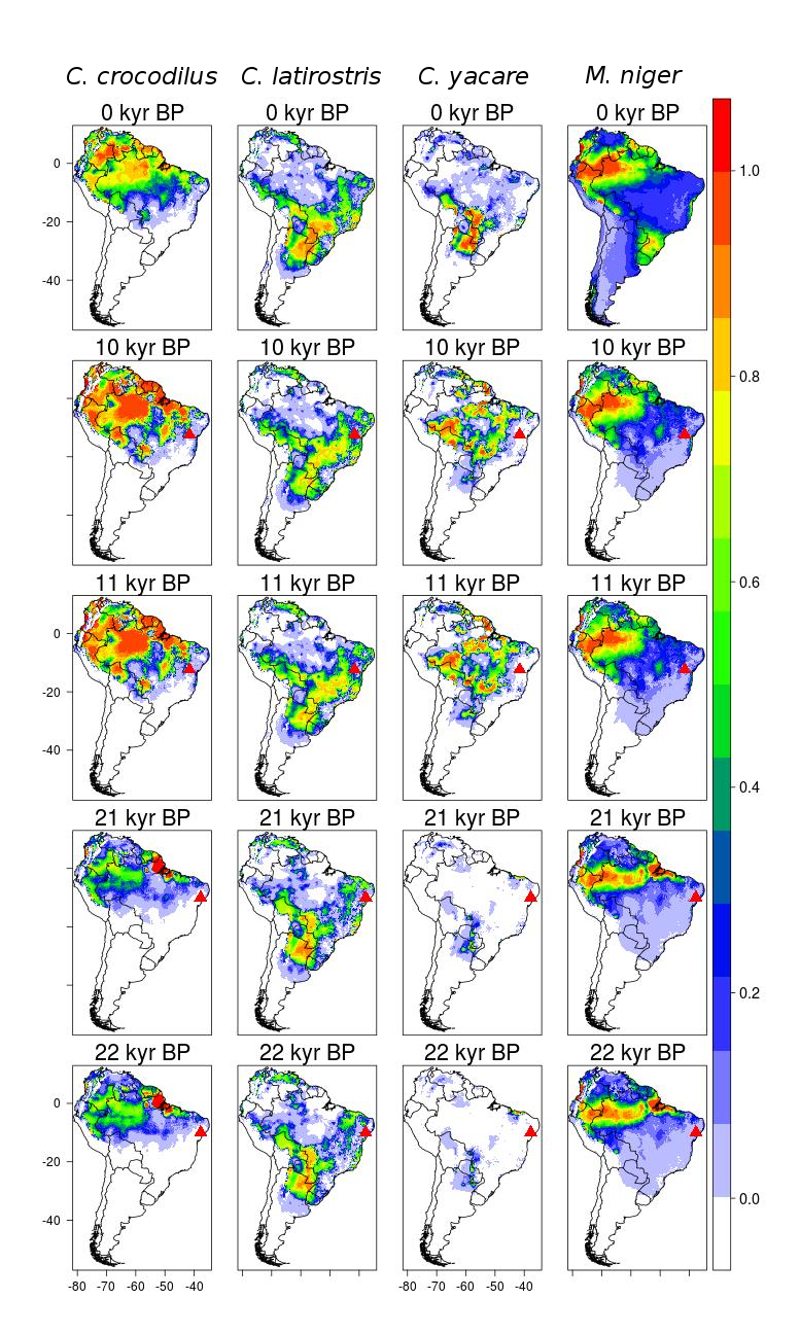

Supplement: S3 Fig — Complete results of our simulations with GLM algorithm, for suitability distribution of Caiman crocodilus, Caiman latirostris, Caiman yacare and Melanosuchus niger on the Neotropics. The blue triangle indicates the points where the fossil were recorded. (TIFF) [file pone.0194725.s004.tiff]

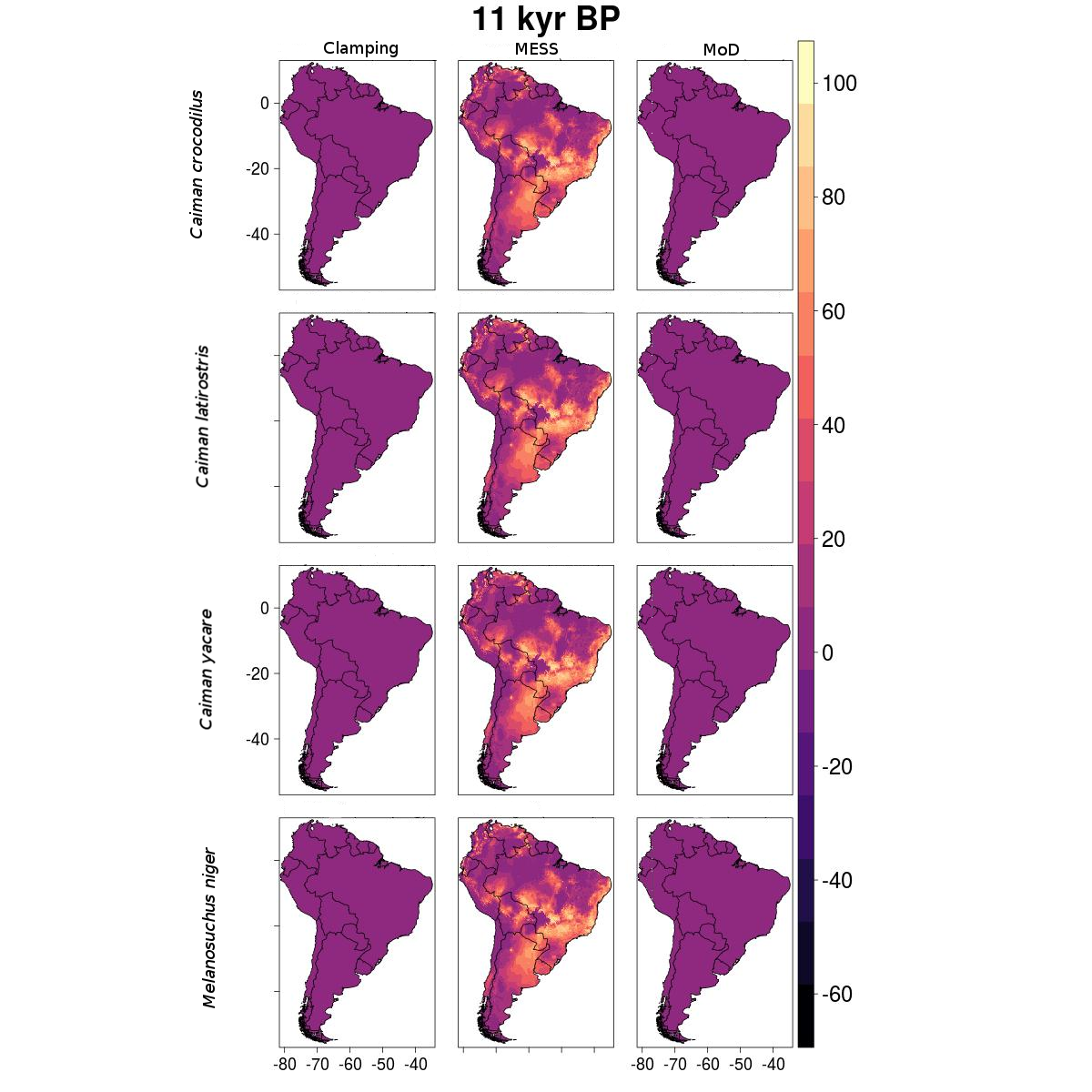

Supplement: S4 Fig — Maxent outputs for Clamping, MESS and MoD, comparing current and 11 kyr BP climates. Results show that do not occur non-analogue climates for such time period, considering the environmental data employed (mean temperature of the warmest and the coldest quarters, and total precipitation of the driest and wettest quarters). (TIFF) [file pone.0194725.s005.tiff]

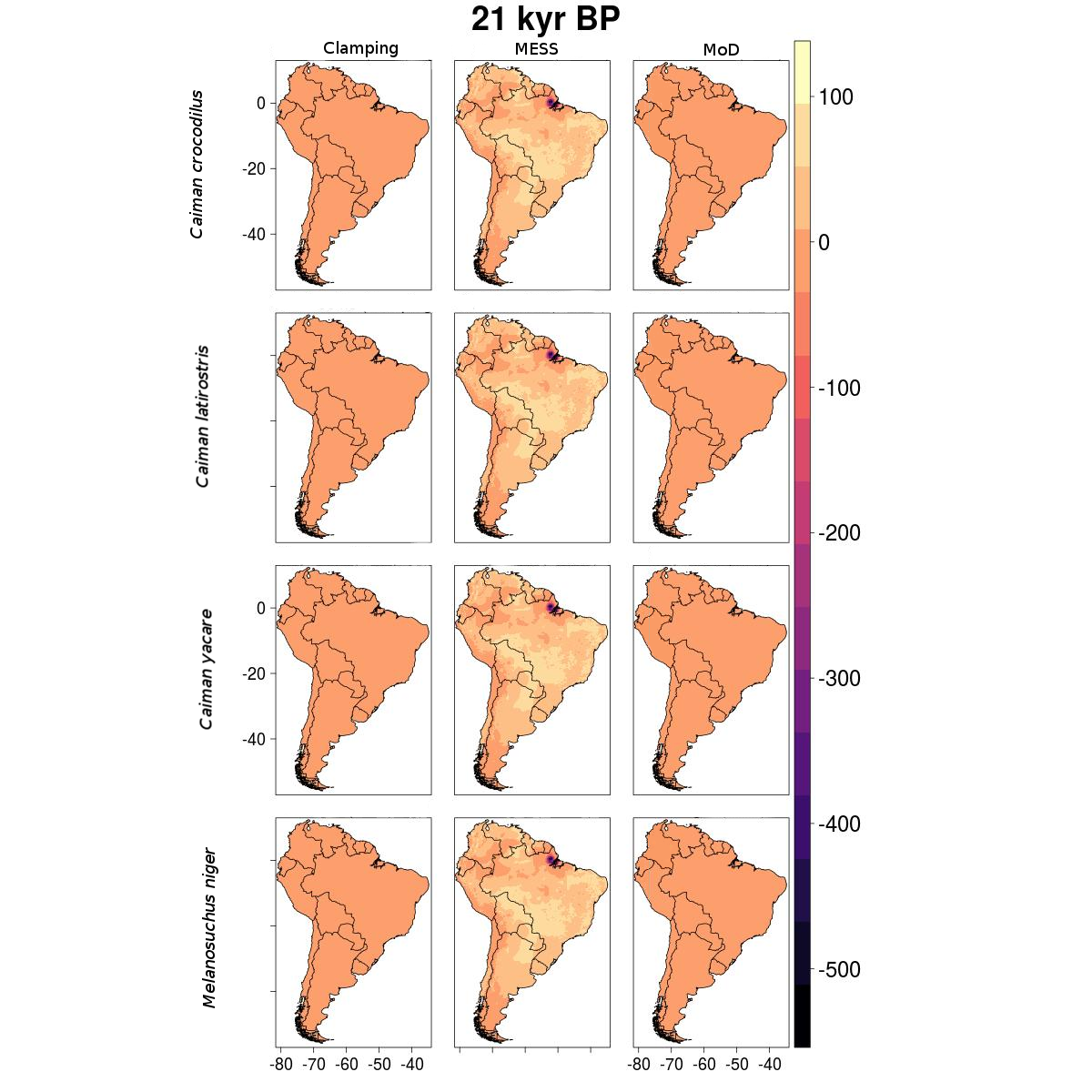

Supplement: S5 Fig — Maxent outputs for Clamping, MESS and MoD, comparing current and 21 kyr BP climates. Results show that do not occur non-analogue climates for such time period, considering the environmental data employed (mean temperature of the warmest and the coldest quarters, and total precipitation of the driest and wettest quarters). (TIFF) [file pone.0194725.s006.tiff]

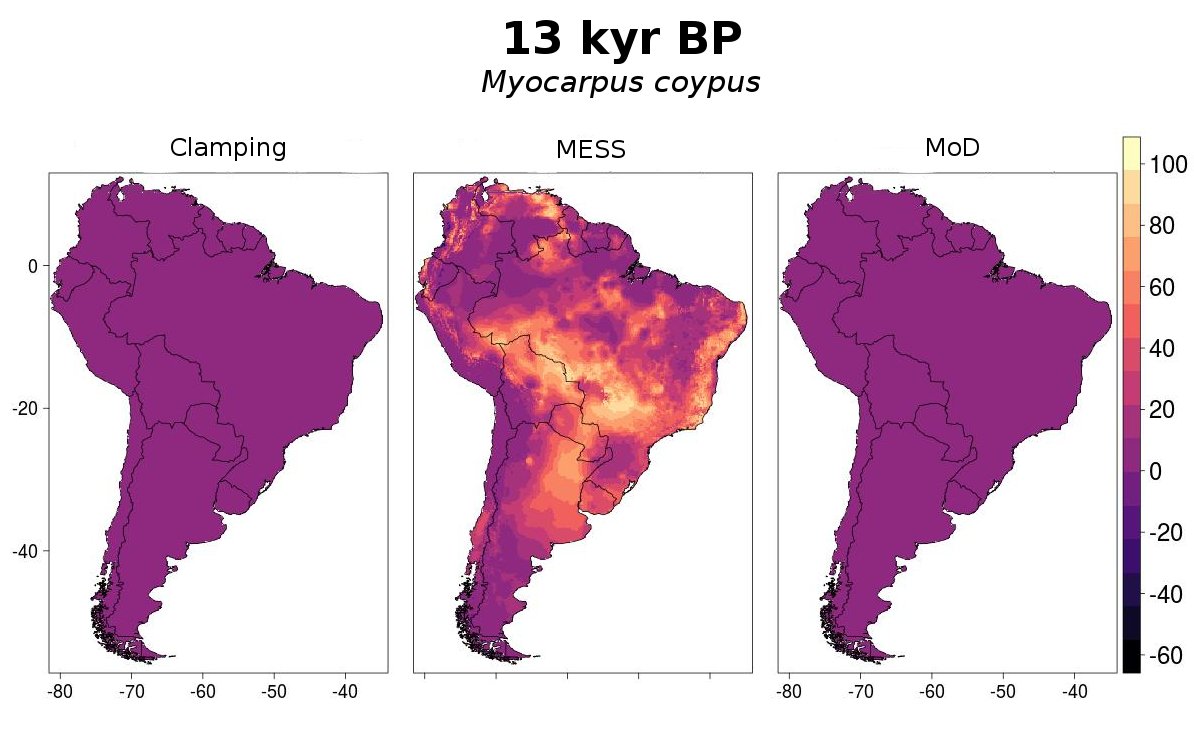

Supplement: S6 Fig — Maxent outputs for Clamping, MESS and MoD, comparing current and 13 kyr BP climates. Results show that do not occur non-analogue climates for such time period, considering the environmental data employed (mean temperature of the warmest and the coldest quarters, and total precipitation of the driest and wettest quarters). (TIFF) [file pone.0194725.s007.tiff]

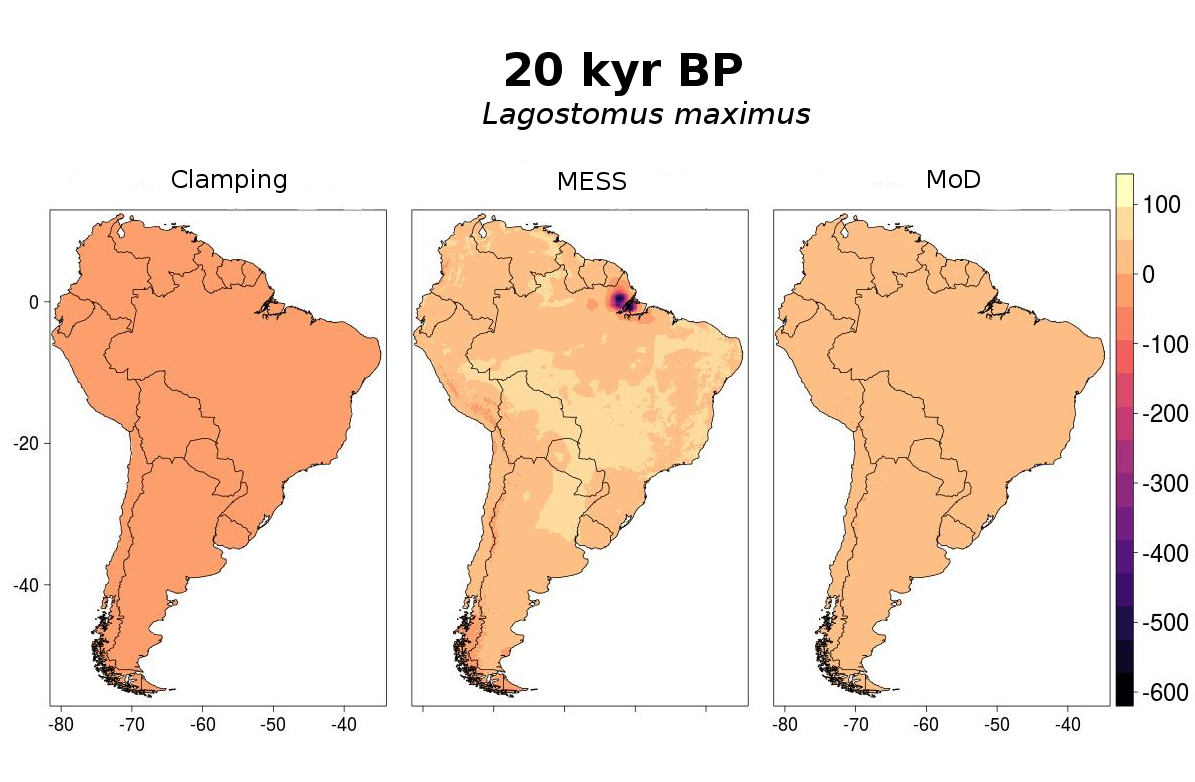

Supplement: S7 Fig — Maxent outputs for Clamping, MESS and MoD, comparing current and 20 kyr BP climates. Results show that do not occur non-analogue climates for such time period, considering the environmental data employed (mean temperature of the warmest and the coldest quarters, and total precipitation of the driest and wettest quarters). (TIFF) [file pone.0194725.s008.tiff]
